# Photomorphogenesis in the Picocyanobacterium *Cyanobium gracile* Includes Increased Phycobilisome Abundance Under Blue Light, Phycobilisome Decoupling Under Near Far-Red Light, and Wavelength-Specific Photoprotective Strategies

**DOI:** 10.3389/fpls.2021.612302

**Published:** 2021-03-18

**Authors:** Gábor Bernát, Tomáš Zavřel, Eva Kotabová, László Kovács, Gábor Steinbach, Lajos Vörös, Ondřej Prášil, Boglárka Somogyi, Viktor R. Tóth

**Affiliations:** ^1^Balaton Limnological Institute, Centre for Ecological Research, Tihany, Hungary; ^2^Centre Algatech, Institute of Microbiology of the Czech Academy of Sciences, Třeboň, Czechia; ^3^Global Change Research Institute, Academy of Sciences of the Czech Republic, Brno, Czechia; ^4^Institute of Plant Biology, Biological Research Centre, Eötvös Loránd Research Network, Szeged, Hungary; ^5^Institute of Biophysics, Biological Research Centre, Eötvös Loránd Research Network, Szeged, Hungary; ^6^Cellular Imaging Laboratory, Biological Research Center, Eötvös Loránd Research Network, Szeged, Hungary

**Keywords:** cyanobacteria, photosynthesis, light-quality acclimation, pigment composition, imbalance

## Abstract

Photomorphogenesis is a process by which photosynthetic organisms perceive external light parameters, including light quality (color), and adjust cellular metabolism, growth rates and other parameters, in order to survive in a changing light environment. In this study we comprehensively explored the light color acclimation of *Cyanobium gracile*, a common cyanobacterium in turbid freshwater shallow lakes, using nine different monochromatic growth lights covering the whole visible spectrum from 435 to 687 nm. According to incident light wavelength, *C. gracile* cells performed great plasticity in terms of pigment composition, antenna size, and photosystem stoichiometry, to optimize their photosynthetic performance and to redox poise their intersystem electron transport chain. In spite of such compensatory strategies, *C. gracile*, like other cyanobacteria, uses blue and near far-red light less efficiently than orange or red light, which involves moderate growth rates, reduced cell volumes and lower electron transport rates. Unfavorable light conditions, where neither chlorophyll nor phycobilisomes absorb light sufficiently, are compensated by an enhanced antenna size. Increasing the wavelength of the growth light is accompanied by increasing photosystem II to photosystem I ratios, which involve better light utilization in the red spectral region. This is surprisingly accompanied by a partial excitonic antenna decoupling, which was the highest in the cells grown under 687 nm light. So far, a similar phenomenon is known to be induced only by strong light; here we demonstrate that under certain physiological conditions such decoupling is also possible to be induced by weak light. This suggests that suboptimal photosynthetic performance of the near far-red light grown *C. gracile* cells is due to a solid redox- and/or signal-imbalance, which leads to the activation of this short-term light acclimation process. Using a variety of photo-biophysical methods, we also demonstrate that under blue wavelengths, excessive light is quenched through orange carotenoid protein mediated non-photochemical quenching, whereas under orange/red wavelengths state transitions are involved in photoprotection.

## Introduction

To be able to survive in ever-changing environments is a critical biological need, and survival/acclimation strategies rely on complex regulatory processes. Sunlight, which is the most important environmental factor for photoautotrophs, and the energy source for most life on Earth, changes continuously – seasonally, daily and also on more rapid time scales. Temporary and spatial changes in light intensities are often accompanied by spectral changes in the available light both in terrestrial and aquatic habitats. In aquatic environments, photosynthetic microorganisms at the surface of the water column are exposed to intense, red-enriched light, while the spectra of available light at moderate or high depth are dominated by green and blue light (or blue light alone). In optically complex environments, such as in shallow waters, the spectrum of available light is affected by the concentration of organic and/or inorganic particles and by the reflection from the lake/sea floor (Maritorena et al., 1994; Ackleson, 2003). Photosynthetic macro- and microorganisms have developed several molecular mechanisms to respond to changes in light quantity and quality to protect themselves from damage by excess light and to optimize their photochemical efficiency at sub-optimal light conditions.

The central features of oxygenic photosynthesis are two sequentially coupled photosystems, photosystem II (PSII) and photosystem I (PSI), located in the thylakoid membrane and connected by the intersystem electron transport chain. Photoautotrophs must regulate the number of light quanta reaching the reaction centers and also light energy distribution between the two photosystems. In the absence of such regulation, the electron transport chain can become under- or oversaturated; while the former involves sub-optimal photosynthetic performance, the latter promotes formation of triplet chlorophyll (Chl) (primarily triplet P680, primary donor of PSII) and, in turn, reactive oxygen species that cause severe damage to cellular components. Adjusting the light-harvesting antenna size and composition, and tuning PSII/PSI ratios according to incident light is a general phenomenon in long-term light acclimation (Fujita et al., 1994; Fujita, 1997; Dietzel et al., 2008).

Various algae and cyanobacteria are able to sense and respond to spectrally variable ambient light conditions (Fujita et al., 1985; Rockwell et al., 2014; Wiltbank and Kehoe, 2016). In cyanobacteria, specific photosensory proteins, cyanobacteriochromes (CBCRs) are employed to detect the spectral quality of irradiance (Fiedler et al., 2004; Ikeuchi and Ishizuka, 2008; Rockwell and Lagarias, 2010). CBCRs exhibit a broad range of wavelength sensitivities, covering the entire visible and near-UV spectrum. The main CBCR function is the regulation of cyanobacterial chromatic acclimation (CA), the mechanisms responding to changes of the ambient spectral light quality (Gutu and Kehoe, 2012; Montgomery, 2017).

Chromatic acclimation occurs in various freshwater and marine habitats and classified into six different types according to the changes in the pigment composition of the of the cyanobacterial light-harvesting antennae, the phycobilisomes (PBS) (Tandeau de Marsac, 1977; Gutu and Kehoe, 2012). In this classification the two distinct phycobiliproteins (PBPs) of the peripheral PBS rods, phycocyanin (PC) and phycoerythrin (PE) have a central role. Species belonging to Group I fail to exhibit change in either PC or PE content in response to changing light. Group II species can adjust PE levels only: they have high PE content under green and low PE content under red light. Species in Group III perform the most dramatic CA known as “complementary chromatic adaptation” that has been described more than a century ago (Engelmann, 1883). They vary PE levels in a similar way as in group II, but, in addition, adjust PC levels to reverse direction, i.e., increase it under red and decrease it under green light. The fourth type of CA responds to blue and green light (Palenik, 2001), and it is specific to deep marine environments (typical representatives are various *Synechococcus* strains). In CA4, similar to CA1, there are no significant changes in PE or PC levels; however, ratios of the two PE-bound chromophores, phycourobilin (blue light absorbing) and phycoerythrobilin (green light absorbing), do change (Everroad et al., 2006). CA5 and CA6 are involved in acclimation to far-red light and involve specific pigments such as chlorophyll *d* and chlorophyll *f* (Sanfilippo et al., 2019).

Although members of CA Group I, same as CA-incapable strains, do not alter their PC and/or PE levels in response to changing light colors, they do sense light color and perform photomorphogenesis (Fujita et al., 1985, 1987, 1994; Fujita and Murakami, 1987; Fujita, 1997; Fiedler et al., 2004; Bernstein et al., 2014). These studies revealed changes in growth rates, and abundance and stoichiometry of photosystems, cytochrome *b_6_f*, and PBSs in response to the color of growth light. However, most of these investigations are restricted only to a few (most often: two) selected growth lights (e.g., PBP and Chl *a* light), rather than covering the entire visible range. Very recently, Luimstra and her co-workers systematically investigated the paradox, why cyanobacteria utilize blue light much less efficiently than orange or red light (Luimstra et al., 2018) growing *Synechocystis* sp. PCC 6803 under blue (425 nm), orange (625 nm) or red (660 nm) light. They combined growth rate determinations, O_2_-yield measurements and UV-Vis, and fluorescence spectroscopy measurements and found an imbalance between PSII and PSI and a related energy deficiency at PSII in cultures grown under blue light.

In this work, we comprehensively study the photomorphogenesis of the unicellular cyanobacterium *Cyanobium gracile* (a strain without any previous record of CA) over the full visible light range from violet to far red light, using nine monochromatic growth lights. We characterize long-term acclimation strategies in terms of pigment composition, light harvesting and energy distribution, redox homeostasis, and cellular plasticity. *C. gracile* naturally occurs in turbid freshwater shallow lakes with an optically complex environment, like Lake Balaton (Felföldi et al., 2011). However, wavelength-specific acclimation strategies in this strain have not been studied up to date. We observed unusual fluorescence properties of *C. gracile* cells when cultivated under certain wavelengths, similar to those found in strong-light exposed *Synechocystis* with high PBS emission (Tamary et al., 2012). The phenomenon was the most pronounced in cells grown under 687 nm light. We interpreted these properties as a signature of substantial excitonic decoupling induced by continuous weak light under certain physiological conditions. This suggests that suboptimal photosynthetic performance of *C. gracile* cells grown under near far-red light is due to a solid redox- and/or signal-imbalance, which leads to the activation of this light acclimation process. In addition, we found wavelength-specific changes in cell morphology, pigment content, photoprotection, and photosynthetic efficiency, suggesting a high level of light utilization plasticity and previously unrecognized light acclimation strategies in *C. gracile*.

## Materials and Methods

### Strain and Culture Conditions

**FIGURE 1:**
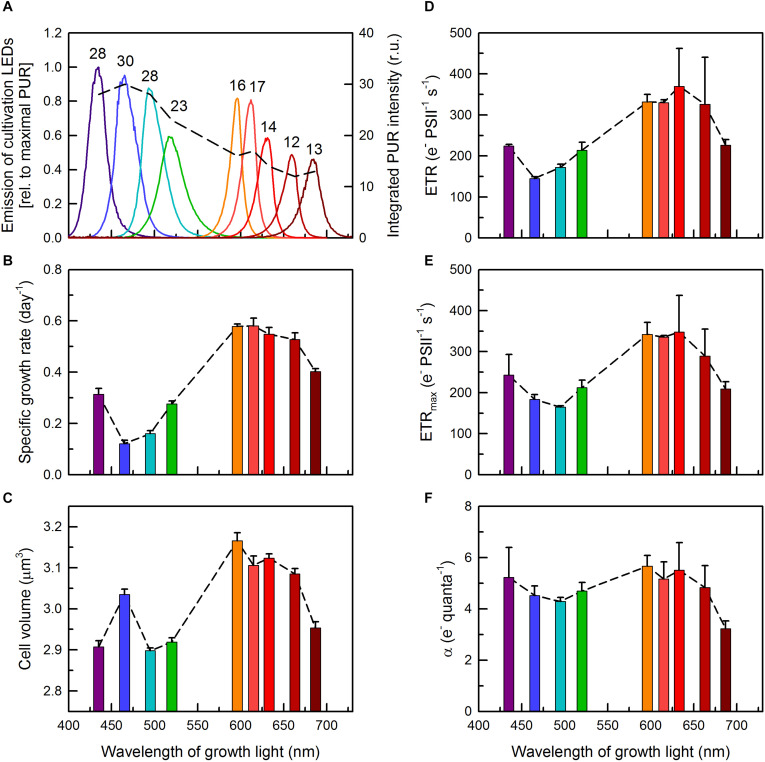
**(A)** Emission spectra of the LEDs used for cultivation of *C. gracile* ACT 1026 (solid lines, left axis). The areas under the individual spectra correspond with photosynthetically usable radiation (PUR; dashed line and numbers on top, right axis) based on the UV-Vis spectra of the *C. gracile* cells grown under monochromatic lights (as detailed in Figure 2B). Growth rates **(B)**, cellular volumes **(C)** and ETR parameters **(D–F)** were determined for *C. gracile* during semi-continuous cultivation under monochromatic lights as shown in panel **(A)**. ETR **(D)**, ETR_max_ (E) and α **(F)** were determined by SP analysis of the corresponding fluorescence induction curves [**(D)**; see also Supplementary Figure 6] and rapid light curves **(E,F)** using 625 nm ML and AL. (For other ML’s and AL’s, see Supplementary Figure 1.) Values of three biological replicates were averaged; standard errors are indicated as error bars in panels **(B–F)**. Dashed lines in panels **(A–F)** represent the trend lines.

The *Cyanobium* strain was isolated from Lake Fertő, Austria, and is being deposited as strain ACT 1026 at the Algal Collection Tihany, Hungary. Its 16S rRNA nucleotide sequence shows 100% similarity to that of *Cyanobium gracile* sp. PCC 6307 (Rippka and Cohen-Bazire, 1983; Felföldi et al., 2011). Cells were grown in Erlenmeyer flasks at 24°C in liquid BG11 medium (Stanier et al., 1971) as semi-continuous batch cultures, exposed to 25 μmol photons m^–2^ s^–1^ diffuse light, placed onto a home-built cultivation apparatus with monochromatic light emitting diodes (LEDs), using a 14:10 h light-dark regime. Nine different growth lights, from 435 to 687 nm, were applied, using the following power LEDs: FD-34UV-Y1 (peak wavelength: 435 nm), FD-3B-Y1 (465 nm), FD-32G-Y1 (495 nm), FD-3G-Y1 (520 nm), FD-3Y-Y1 (596 nm), FD-3A-Y1 (615 nm), FD-3R-Y1 (633 nm), FD-333R-Y1 (663 nm), FD-34R-Y1 (687 nm) (Shenzhen Fedy Technology Co., Ltd., Shenzhen, China). LED emission spectra, corrected by the photosynthetically usable radiation, PUR, accessible to the corresponding cultures, are shown in detail in Figure 1A. Cultures were shaken manually once a day. By considering the growth rates of the cultures at selected wavelengths (see section “Results”), cultures were diluted to an appropriate cell density in advance (usually 4 days before the measurements), resulting in OD_750_ = 0.2 at the day of the measurements (see the next section for further details). Such setup provided appropriate (long-term) light acclimation and secured keeping the cells in the exponential growth phase.

### OD Determination, Cell Counting and Determination of Growth Rates

Optical density of the cultures was determined at 750 nm using a U2900 Hitachi double beam spectrophotometer (Hitachi, Tokyo, Japan). Cells were counted and size-determined using a Cellometer Auto M10 (Nexcelom Bioscience, Lawrence, MA, United States) and an ImageStream MkII imaging flow cytometer (Amnis Corp., Seattle, WA, United States), respectively. Right after harvesting, cells were treated with 2% formaldehyde and incubated for 10 min at room temperature. The fixed cells were stored at –20°C until further use (up to 3 weeks). Prior to further analysis, samples were thawed slowly (during ∼1 h) at room temperature. To discriminate *C. gracile* cells from debris/bacterial contamination, 5 μL of SYBR Green I was added to each sample. During cytometric analysis, gating of the measured populations was applied to discriminate (i) focused objects (*via* combined use of RMS gradient and Threshold Mask features of the IDEAS instrumental software) and (ii) round objects (width/length ratio from 0.9 to 1.0). The imaging flow cytometer was calibrated using non-fluorescent microspheres (1–15 μm, Thermo Fisher Scientific, Waltham, MA, United States), and the results were validated with an Axio Imager 2 light microscope (Carl Zeiss, Oberkochen, Germany). During cytometric analysis, Chl *a* and PBS autofluorescence (excitation: 642 nm, detection: 642–745 nm) were also recorded to validate selection of the cells within all measured objects. Bright field images were used for cell size analysis; and mean cell volumes in the cultures were calculated assuming spherical cell shapes. At OD_750_ = 0.2 cell densities varied between 3.5 and 7.7 × 10^7^ cells mL^–1^. Growth rates were determined by fitting the OD_750_ values by an exponential function.

### Photosynthetic Activity Measurements

**FIGURE 2:**
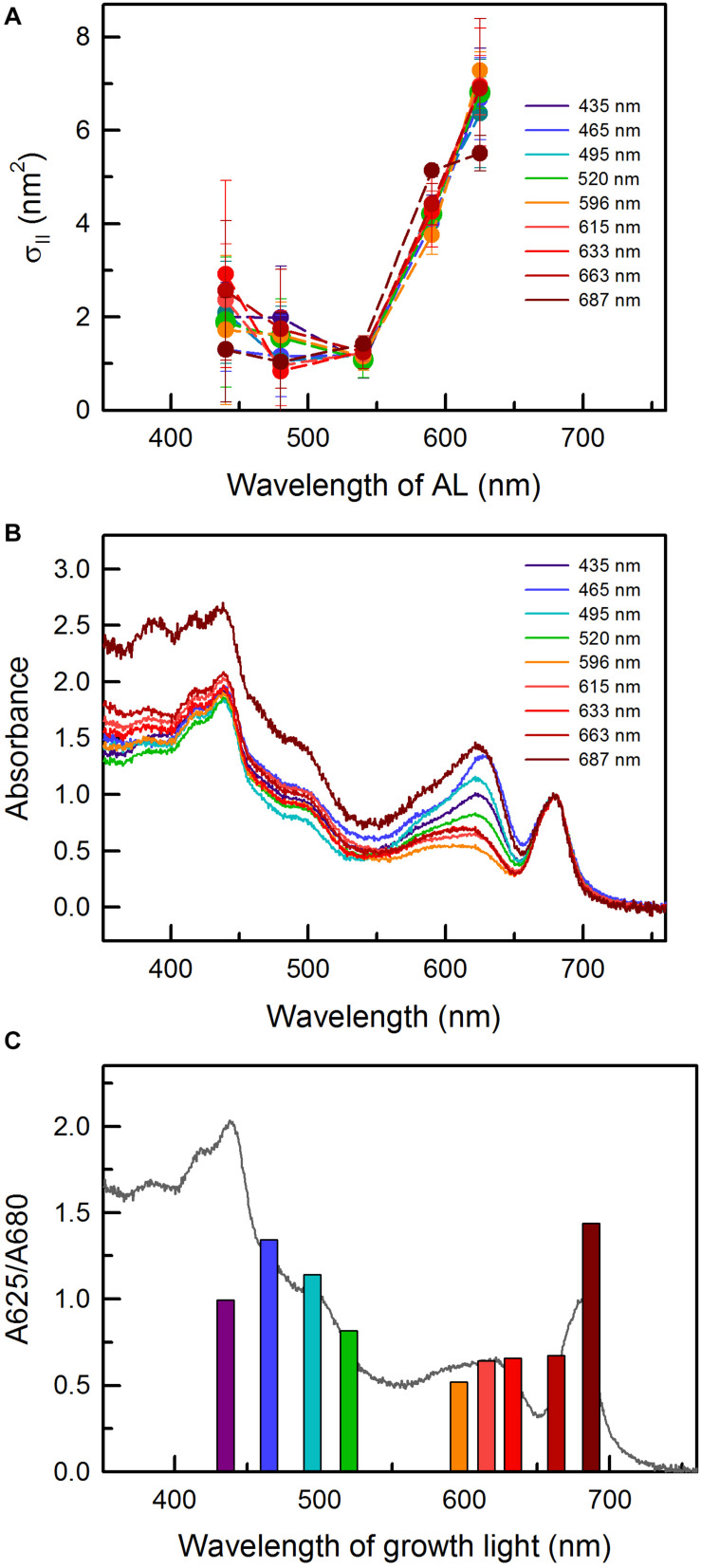
**(A)** Functional absorption cross-section of PSII, σ_II_, as a function of actinic light (AL) wavelength in *C. gracile* sp. ACT 1026 cells grown under monochromatic lights (as detailed in Figure 1A). Values of three biological replicates were averaged; standard errors are indicated as error bars. **(B)** Absorbance spectra of *C. gracile* grown under particular monochromatic lights. Spectra were baseline corrected and normalized to the 680 nm Chl *a* Q-band. Each spectrum represents the average of three biological replicates; error intervals are not shown for clarity. **(C)** A625/A680 ratios as a function of growth light. Averaged absorbance spectrum of cultures grown under 615 nm light is shown as a reference. The legends in panel **(A,B)** indicate the wavelengths of growth lights.

Photosynthetic performance of *C. gracile* under actinic light (AL) illumination was probed using a Multi-Color-PAM (MC-PAM; Walz, Effeltrich, Germany) by recording fluorescence induction, OJIP and rapid light curves. Photosynthetically relevant parameters [e.g., electron transport rates (ETR)] were determined using saturating pulse (SP) analysis under five different (default) AL and measuring light (ML) wavelengths of the instrument: 440, 480, 540, 590, and 625 nm. In all cases, AL and ML were set to the same color. Importantly, for all but one (Figure 2A) figures in the main text 625 nm AL and ML are used and additional data (when relevant) are provided in the Supplementary Material. Fluorescence induction curves with 5 min AL period were recorded after 20 min of dark acclimation; AL intensity at each wavelength was set to 100 μmol photons m^–2^ s^–1^, which (moderate) light intensity was sufficiently high to induce fluorescence transients. AL intensities during rapid light curve recordings stepwise increased from 0 to 1300–1700 μmol photons m^–2^ s^–1^ (depending on the AL used) through 20 individual steps of 30 s duration each, following default protocol settings defined by the manufacturer. Here, besides the first (dark) step, no extra dark acclimation was applied. Functional absorption cross-section of PSII, σ_II_, was determined after 20 min of dark acclimation, using the default script “Sigma1000cyano.”

Fast fluorescence induction kinetics (OJIP) was recorded by *Fast Kinetics* MC-PAM feature after 360 s of dark acclimation, using *FastKin_Def* trigger settings and saturation pulses of five wavelengths (440, 480, 540, 590, and 625 nm) and of maximal intensity up to 20 000 μmol photons m^–2^ s^–1^.

### Absorption (UV-Vis) Spectroscopy

Whole cell absorption spectra were recorded by Unicam UV 500 scanning spectrophotometer (Thermo Spectronic, Cambridge, United Kingdom) using a pair of 1-mm transparent quartz cuvettes placed into the outer cuvette holder of the instrument (with shorter optical path length, which is beneficial for recording absorption spectra of cell suspensions with substantial light scattering). In order to obtain a high signal-to-noise ratio, 120 nm/min scan speed and 2.0 nm bandwidth were applied.

### Low Temperature Fluorescence Emission and Excitation Spectroscopy

A total of 10 mL of cell suspension was filtered through a 25 mm GF/B glass microfiber filter (Whatman, Maidstone, United Kingdom) under dim light. Moist, oval-shaped fragments (∼1 cm × 0.3 cm) were cut using a deformed cork drill stored at −80°C prior to use (up to 6 months). For measurements, the fragments were placed into the cavity of a copper finger of a home-made apparatus (Prášil et al., 2009) and were immersed into a transparent Dewar flask filled with liquid nitrogen. A total of 77 K fluorescence emission spectra were recorded using an SM-9000 spectrophotometer (Prášil et al., 2009; Photon System Instruments [PSI], Brno, Czechia) upon excitation with 457 or 534 nm light (20 nm bandwidths) for Chl and phycobilin excitation, respectively. Three individual spectra of three replicates (9 in total) were recorded, baseline-corrected and averaged.

Fluorescence excitation spectra were recorded similarly using an Aminco-Bowman AB2 Luminescence Spectrometer (SLM Spectronic Instruments, Rochester, NY, United States) with fluorescence emission recorded at either 655, 685, 728, 745, or 765 nm. Both the excitation and emission bandwidths were set to 4 nm. Three individual traces of each sample were averaged, and the raw data were divided by the signal from the reference photomultiplier tube to provide a first-order quantum correction.

### Pigment Analysis

Phycobiliprotein content was estimated by semi-quantitative analysis of the whole-cell absorption spectra, based on the method of Bennett and Bogorad (1973), and followed by normalizing the phycobilisome content to the respective absorbance at 750 nm. Although the extinction coefficients determined by Bennett and Bogorad (1973) are specific for 1 cm optical path length, due to the linear dependence of the absorbance on path length, they can also be used for semi-quantitative determination e.g., in 1 mm cuvettes.

Chlorophyll and carotenoid contents of the samples were determined by high-performance liquid chromatography (HPLC). Cells from 10 mL cultures aliquots were harvested on a Whatman glass microfiber filter (GF/B; ∅ 25 mm) and were kept frozen at –80°C until extraction (for less than 2 weeks). Soluble pigments were extracted by adding 500 μL of pure acetone and concomitant shaking for 30 min at 1,000 rpm in the dark at 25°C. Insoluble constituents were span down at 11 500 × *g* at 4°C for 10 min, and the supernatant was passed through a polytetrafluoroethylene (PTFE) syringe filter with a pore size of 0.2 μm.

Carotenoid composition of the acetonic extracts was analyzed using a Shimadzu Prominence HPLC system (Shimadzu, Kyoto, Japan) consisting of two LC-20AD pumps, a DGU-14A degasser, a SIL-30A automatic sample injector, a column thermostat and a Nexera X2 1024-element photodiode-array detector. Chromatographic separations were carried out on a Phenomenex Synergi Hydro-RP 250 × 4.6 mm column with a particle size of 4 μm and a pore size of 80 Å. 20 μL aliquots of acetonic extract were injected to the column and the pigments were eluted by a linear gradient from solvent A (acetonitrile, water, trimethylamine; in a ratio of 9:1:0.01) to solvent B (ethyl acetate). The gradient from solvent A to solvent B was run from 0 to 25 min at a flow rate of 1 mL/min. The column temperature was set to 25°C. Eluates were monitored in a wavelength range of 260 to 750 nm. Pigments were identified according to their retention time and absorption spectrum and quantified by integrated chromatographic peak area recorded at the wavelength of maximum absorbance for each pigment using their corresponding absorption coefficient.

### Confocal Microscopy

Confocal microscopy were performed using a Leica DMi8 confocal microscope (Leica, Wetzlar, Germany) equipped with a HC PL APO CS2 63×/1.4 oil objective and a TD 488/552/638 main beam splitter. Cells were immobilized on a thin (<1 mm) layer of solid BG11 agar and placed upside down onto a cover slide. Chl *a* and PBS autofluorescence were excited using the 488 and 638 nm lasers, respectively, and were detected over the 690–790 nm and 650–680 nm spectral windows with PMT detectors. The microscope provided a 2048 × 2048 pixel resolution with a 183.65 × 183.65 μm picture size (90 nm/pixel). Images were processed using the ImageJ (1.52c) open source Java image processing program (Schneider et al., 2012). In order to exclude low intensity background images, fluorescence channels were separated and images were converted into a 32 bit floating point format using a macro. The mean fluorescence intensity of the cells was calculated as an average intensity above the threshold. For statistical analysis, intensity profiles for both channels were evaluated along the cellular cross sections and fluorescence intensities of pixels positioned with identical distances from the geometrical center of cells were averaged. Ratios of the fluorescence intensities at centers of cells (first values of the folded datasets) and intensities of membrane regions (corresponding maxima) were calculated.

## Results

### Growth Rates, Electron Transport Rates, and Overall Fitness of the Cell Cultures

Growth rates of *C. gracile* was found to be wavelength-dependent. Similarly to other cyanobacteria (Wyman and Fay, 1986; Luimstra et al., 2018), *C. gracile* grows slowly under violet, blue and green light, and relatively fast under red light. Plotting the growth rates against wavelength of growth light shows the lowest rates at around 465–495 nm (μ = 0.12–0.16 d^−1^), and a broad plateau of high growth rates (μ = 0.52–0.58 d^−1^) from 596 to 663 nm (Figure 1B). The observed slow growth at the blue-blue green spectral region was accompanied by low maximal culture densities (data not shown). Reaching the far-red region (687 nm) resulted in considerably slower growth rates again (Figure 1B). This pattern does not fit the changes in PUR (in fact, it was more or less the opposite; Figure 1A), hence, cannot be explained by changes in light quanta absorbed (see section “Discussion” for further details).

Interestingly, growth rates correlated considerably with cell volumes, i.e., the cells were smaller in most cultures grown under violet to green and under near far-red light, and larger in cultures grown under orange to red light (Figure 1C), albeit the relative difference in cell volumes was much smaller (up to 9%) than the corresponding difference in growth rates (up to 4.8-fold). Nevertheless, the insufficient microbial growth under sub-optimal light conditions was accompanied by smaller cell volumes. To elucidate such dramatic wavelength dependence of growth, we further probed how pigment levels, light capture and distribution, and other bio-energetically relevant parameters did also change (or did not change) with varying growth light.

In order to address the latter issue, steady-state and maximal PSII-mediated electron transport rates (ETR(II) and ETR(II)_max_, respectively) were determined by SP analysis of the corresponding fluorescence induction curves and rapid light curves (Figures 1D,E, respectively). Irrespective of the applied AL color, the light quality dependences of both parameters were similar to those of the growth rates (Figure 1B and Supplementary Figure 1), showing, not surprisingly, a tight correlation between photosynthetic performance and growth characteristics. Nevertheless, the relatively large variation in growth rates (4.8-fold, see above) was accompanied by a smaller variation in ETR values (2.1-fold), indicating a non-proportional (linear) relationship between ETR and growth rates. Noteworthy, steady-state ETR(II), apparently, exceeded ETR(II)_max_ in some cases (compare Figures 1D,E), which, possibly, was due to the different approach applied at determining these parameters (see section “Materials and Methods”) and also state transitions occurred during rapid light curves (see section “Photosynthetic Performance” for details). Slopes of the ETR light curves in the initial, light-limited phase, α (Figure 1F) showed similar light quality dependence as the other, above mentioned parameters; however, performed a much smaller overall variability (i.e., photosynthetic performance in the light-limited phase did only weakly depend on growth light). The lowest α value (i.e., theoretical maximal ETR at any light intensity) belonged to the cultures acclimated to 687 nm light, showing an impaired electron transport in these cultures.

### Light Absorption and Pigment Composition

The AL-color dependency of the functional absorption cross-section of PSII, σ_II_, showed a similar pattern to that of *Synechocystis* sp. PCC 6803 (Schreiber et al., 2012): low σ_II_ values up to 540 nm, and a remarkable increase above this wavelength (Figure 2A). The AL-color dependency of σ_II_ was irrespective of the growth light with only one exception: the *C. gracile* cells grown under 687 nm showed significantly relatively smaller cross section of PSII as compared to other cultures when 625 nm (i.e., red) AL was applied (one-way ANOVA followed by Tukey’s HSD *post hoc* test; *p* < 0.05). This smaller functional antenna size may explain the lower α of these cultures (see previous paragraph).

**FIGURE 3:**
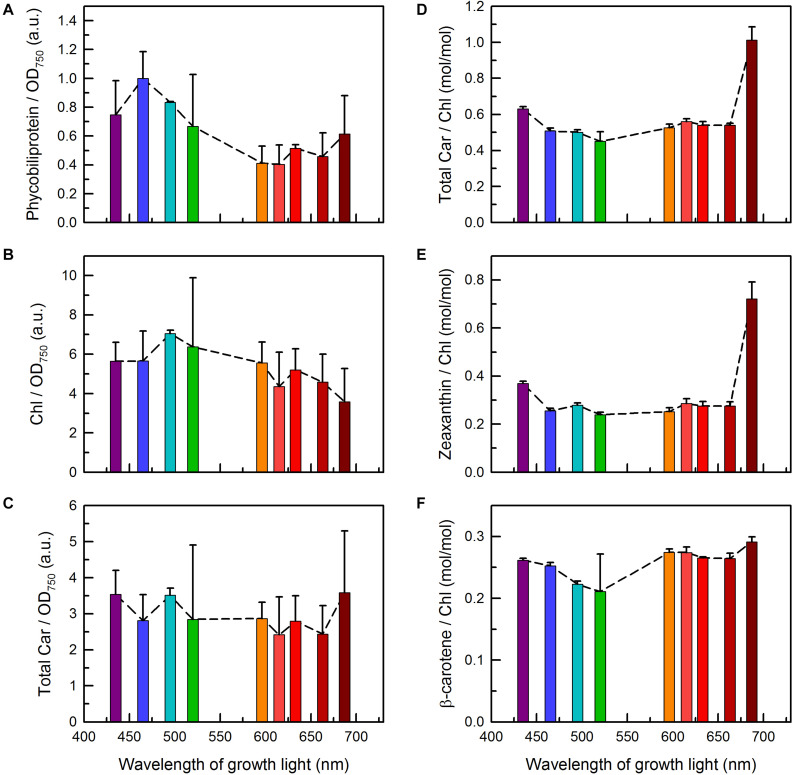
Pigment contents in *C. gracile* sp. ACT 1026 cultures grown under monochromatic lights (as detailed in Figure 1A). Total phycobiliprotein content of the cultures **(A)** was determined based on the absorbance spectra shown in Figure 2B, while the levels of Chl *a* and carotenoids **(B–F)** were determined by HPLC. Pigment levels were normalized either to OD_750_ [**(A)**, total phycobiliprotein; **(B)**, Chl *a*; **(C)**, total carotenoid] or to the corresponding Chl *a* concentration [**(D)**, total carotenoid; **(E)**, zeaxanthin; **(F)**, β-carotene]. Phycobiliprotein to Chl *a* ratios are shown in Supplementary Figure 2. Data are expressed as mean ± standard error (*n* = 3). Dashed lines in panels **(A–F)** represent trend lines in both panels.

The minimal growth rates of *C. gracile* at 465 and 495 nm (Figure 1B) can be explained by the combination of ineffective utilization of the blue-blue green light in cyanobacteria (see also Luimstra et al., 2018, and references therein) and the absorbance profile of the cells showing a remarkable drop in Chl absorption at the red side of the Soret band (Figure 2B). In general, the absorbance spectra of *C. gracile* were similar to that of other cyanobacteria (see e.g., Kopečna et al., 2012; Luimstra et al., 2018) with Chl *a* Soret band at 440 nm (together with two shoulders at 387 and 420 nm) and a Q-band at 680 nm, a carotenoid shoulder at 485 nm, and phycocyanobilin absorption peaked at 625 nm. The recorded spectra showed high variability, mainly due to alterations in phycobilin absorption, which displayed strong dependency on the wavelength of the growth light (Figure 2B). Plotting the A625/A680 ratios against growth wavelengths revealed high PBS to Chl ratios over the violet to blue-green region with the highest A625/A680 ratio at around 465–495 nm, as well as at 687 nm, and a low, stable PBS abundance between these wavelengths (Figure 2C). Semi-quantitative analysis of the cellular PBP content showed a similar trend with the same local maxima, however, with about 60% more PBS in the blue part of the spectra as compared to the near far-red region (Figure 3A). These results imply a low Chl content in the 687 nm grown cells, in agreement with direct determination of the Chl *a* levels by HPLC (Figure 3B). Unsurprisingly, normalization of the PBP content to the Chl concentration, revealed the same trend as of the A625/A680 ratios (Figure 2C and Supplementary Figure 2). Besides, the remarkably high absorbance and modified spectral shape of the 687 nm grown cultures in the blue spectral region suggests an elevated carotenoid level in these cells (as compared to Chl *a*), which is also supported by a more intense carotenoid shoulder at 485 nm (Figure 2B; Kopečna et al., 2012). This, again, is well supported by HPLC data (Figures 3C–F).

High-performance liquid chromatography chromatograms of *C. gracile* cells were dominated by three major bands with retention times of 13.7, 16.6, and 20.4 min, originating from zeaxanthin, Chl *a*, and β-carotene, respectively (Supplementary Figure 3; Komárek et al., 1999; note that β-carotene was misidentified as α-carotene that time). In cells grown under blue/blue-green light, Chl *a* allomers, to a certain extent, were also present (Supplementary Figure 3), showing an increased level of reactive oxygen substances, which concurs with a reduced plastoquinone (PQ) level in these cells (see section “Photosynthetic Performance” for details). Quantitative analysis of the pigment composition (Figures 3B–F) revealed remarkable changes in the Chl and total carotenoid level in the cultures grown under 687 nm light, in particular, lowered Chl *a* and enhanced carotenoid levels, respectively (Figures 3B,C). This negative correlation results in a very high ratio of total carotenoids to Chl *a* in these cells (Figure 3D). Lower, but still significant (one-way ANOVA followed by Tukey’s HSD *post hoc* test; *p* < 0.05) increase in total carotenoids to Chl *a* ratio was also found in *C. gracile* cells grown under violet light (435 nm, Figure 3D). In both cases, the enhanced total carotenoid to Chl *a* ratio originated dominantly from the increase of zeaxanthin (Figure 3E). As compared to zeaxanthin, the level of β-carotene showed weaker dependence on the growth light (Figure 3F). However, we found statistically significant decrease (one-way ANOVA followed by Tukey’s HSD *post hoc* test; *p* < 0.05) in β-carotene to Chl *a* ratio in the cells grown at 495 and 520 nm (Figure 3F), in correlation with the substantial level of allomeric substances in these cultures (see above).

### Excitation of Photosynthetic Pigment-Protein Complexes

**FIGURE 4:**
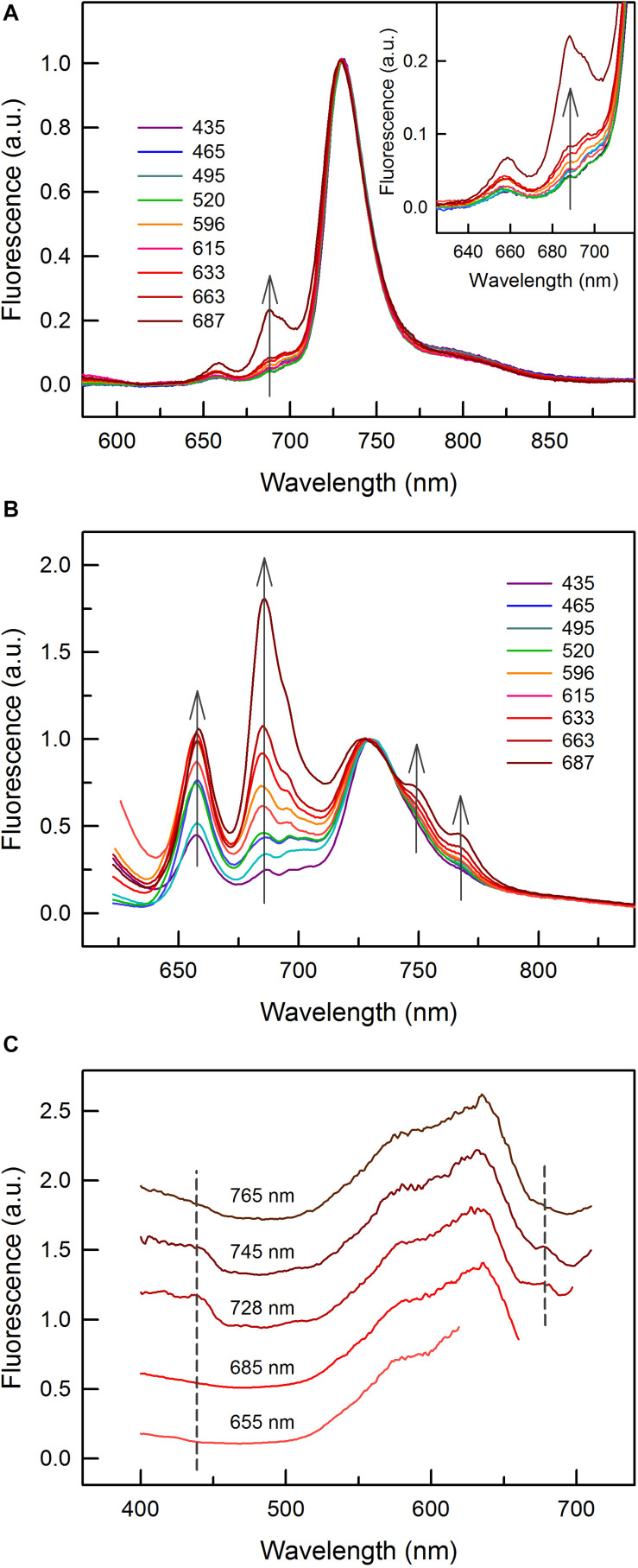
Low temperature (77 K) fluorescence emission and excitation spectra of *C. gracile* sp. ACT 1026. **(A,B)** Fluorescence emission spectra of cells grown under monochromatic lights with 455 nm (Chl *a*) and 590 nm (PBS) excitation, respectively. Three individual spectra of three technical replicates (9 spectra in total for each light condition) were averaged and baseline corrected; error intervals are not shown for clarity. Spectra were normalized to the 685 nm emission. The inset on panel A zooms in the 625 nm – 719 nm spectral region. The gray arrows show the intensification of certain spectral peaks or shoulders with increasing growth wavelengths. **(C)** Fluorescence excitation spectra of the 687 nm grown cells, with various excitation wavelengths as indicated above each spectrum. Three individual spectra were averaged and reference corrected. Spectra were normalized to the maximal signal intensity and shifted vertically for clarity. Dashed lines represent the wavelengths of Chl *a* absorption maxima. The narrower spectra with 655, 685, and 728 nm excitation lights are due to the gap between excitation and emission wavelengths.

Spectral properties of *C. gracile* cells were further examined by low temperature (77 K) fluorescence spectroscopy. Fluorescence emission spectra with 455 nm (Chl *a*) excitation showed three major spectral features: a minor PBP emission at 655 nm; a double emission peak at 685–695 nm, originating from PSII (emitting at both wavelengths) with a variable contribution to the signal by the PBS terminal emitter (emitting at 685 nm only); and a robust PSI emission peaking at 728 nm (Figure 4A), indicating small PSII/PSI ratios over the whole visible range, regardless of the growth wavelength. Nevertheless, increasing the growth wavelength from 435 to 663 nm resulted in a remarkable increase in the PSII/PSI ratio (Figure 4A, main panel and inset). The proportional increase of the 685 and 695 nm emission suggests only a minor contribution of the PBS terminal emitter. Contrary to this, the cells grown under 687 nm light show an intense fluorescence emission at 685 nm and a distorted shape of the 685–695 nm double band, showing a considerable light emission from the PBS terminal emitter.

This feature is much better seen in Figure 4B where fluorescence emission spectra with direct PBS excitation at 590 nm are shown. This series of spectra revealed that PBS decoupling, to a certain extent, took place also at shorter growth wavelengths but it became dominant at 687 nm (Figure 4B). Enhancing fluorescence intensities of the 655 nm emission as compared to the PSI emission at 728 nm are in good accordance with the increasing PSII/PSI ratios (Figure 4A), and rather than showing increasing PBS levels (c.f. Figure 2), they indicate changes in the excitation energy transfer. Besides these, the spectra also show the intensification of two shoulders, at 745 and 765 nm, respectively, with unknown origin. As with increasing growth wavelengths they increase concomitantly with the intensity of the major emission peak of the terminal emitter at 685 nm (Figure 4B), they might have a related origin.

The interpretation of the fluorescence emission spectra of the 687 nm grown *C. gracile* cells is well supported by fluorescence excitation measurements. As expected, the 655 nm PBP fluorescence emission peak can exclusively be excited by photons absorbed by phycobilins (Figure 4C). The same is true for the 685 nm emission with mixed origin, which confirms our assumption that it originated dominantly from PBS in *C. gracile* grown at 687 nm. In contrast, the 728 nm (PSI) emission band can also effectively be excited by 440 nm blue or 680 nm red light, characteristic for Chl absorption (Figure 4C). The shoulders at 745 and 765 nm can also be excited by 440 and 680 nm light, however, to a smaller extent as compared to the 728 nm emission peak. This, again, suggests that both of these shoulders have a PBS-related origin.

**FIGURE 5:**
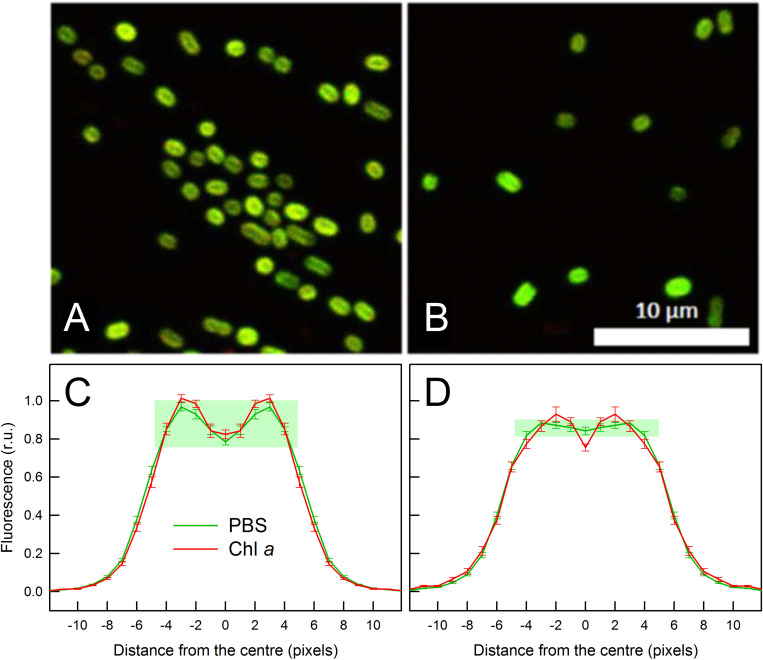
Confocal micrographs **(A,B)** and respective autofluorescence intensity profiles **(C,D)** of *C. gracile* sp. ACT 1026 cells grown under 615 nm **(A,C)** and 687 nm **(B,D)** light. Multicolor images in panels **(A,B)** were composed of PBS (green) and Chl *a* (red) autofluorescence; scale bar is indicated in white. For autofluorescence profiles, fluorescence intensities of 18 **(A,C)** + 10 **(B,D)** = 28 cells were normalized to the integrated intensities along the cross-sectional profile and averaged. Data in panels **(C,D)** are expressed as mean ± standard error; *n* = 36 **(C)** and 20 **(D)**, note the central symmetry of the cells. Distances are expressed in pixel units (1 pixel = 90 nm); pixel Nr. 0 indicates the middle of the cells. The difference between PBS autofluorescence profiles is highlighted by green rectangles in panels **(C,D)**.

We explored the unique fluorescence properties of *C. gracile* cells grown under 687 nm light also by confocal microscopy. Figure 5 shows multicolor confocal micrographs of the 687 nm grown cells (B) together with control cells grown under 615 nm light (A). Both types of cells are oval shaped with 1.80 ± 0.01 μm (615 nm) and 1.78 ± 0.01 μm (687 nm) mean diameter (see also Figure 1B). The control cells grown under 615 nm light showed a typical cellular pattern (Tamary et al., 2012) with intense, ring-shaped autofluorescence from the cell periphery, where thylakoid membranes are located (Figures 5A,C). As images were composed of PBS- (green) and Chl *a* (red) autofluorescence, the green-yellow color indicates that the majority of autofluorescence originated from colocalized Chl *a*- and PBS emitters. Quantitative analysis of the images revealed similar, 77.1 ± 14.2% and 80.6 ± 11.0% Chl *a* and PBS fluorescence intensities, respectively, in the central region of the cells grown at 615 nm as compared to the membrane region (Figure 5C). Contrary to this, the cells grown under 687 nm light showed fluorescence patterns to be more homogenous pattern across the cells and dominated by green color (Figure 5B). This latter indicates increased PBS/Chl *a* fluorescence ratios in these cells, in good agreement with the fluorescence spectroscopy data (Figure 4). Image analysis resulted in 1.27 ± 0.06 and 1.74 ± 0.11 PBS/Chl *a* relative autofluorescence ratios for the 615 nm grown and 687 nm grown cells, respectively. The more homogenous pattern suggests that PBS autofluorescence originated not only from the thylakoid membranes, but also from the cytoplasmic space, which is also supported by image analysis. Here, the spatial distribution of Chl *a* autofluorescence remained unchanged as compared to the 615 nm grown cells (77.6 ± 14.9% fluorescence intensities in the central region vs. the membrane region), while the PBS autofluorescence dispersed across the whole cell profile (94.0 ± 4.0% fluorescence intensities in the central region vs. the membrane region, Figure 5D).

### Photosynthetic Performance

**FIGURE 6:**
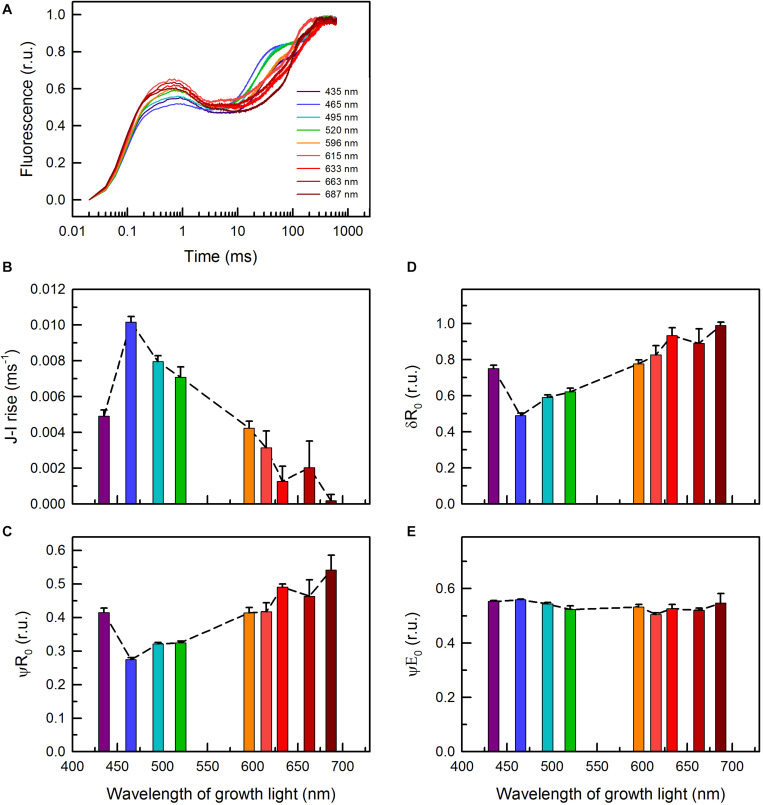
**(A)** OJIP curves of *C. gracile* sp. ACT 1026 cells grown under monochromatic lights (as detailed in Figure 1A) and derived parameters as a function of the wavelength of growth light **(B–E)**. **(B)** Rate of the fluorescence rise during the J-I phase; **(C)** ψR_0_, efficiency with which a PSII trapped electron is transferred to final PSI acceptors; **(D)** δR_0,_ efficiency with which an electron from PQH_2_ is transferred to final PSI acceptors; **(E)** ψE_0_, efficiency with which a PSII trapped electron is transferred from Q_A_^–^ to PQ; the parameters were derived according to Stirbet et al. (2018). Curves on panel **(A)** represent the mean of three biological replicates, normalized to the P level (error intervals are omitted for clarity), while on panels **(B–E)** standard errors and trendlines are indicated as error bars and dashed lines, respectively. In each case, 625 nm ML and saturating pulse was used. Yet, the traces of parameters in panels **(B–E)** were independent of the applied measuring light wavelength (for more details, see Supplementary Figure 4).

**FIGURE 7:**
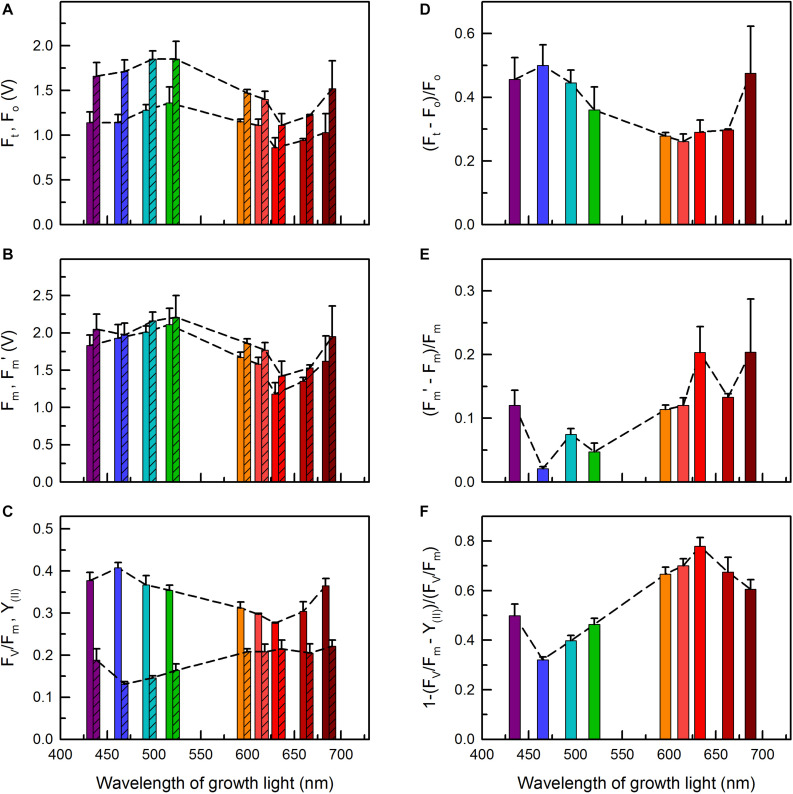
Chl *a* fluorescence parameters of *C. gracile* sp. ACT 1026 cells grown under monochromatic lights (as detailed in Figure 1A) as a function of growth light wavelength. **(A)** F_o_ (open bars) and F_t_ (striped bars); **(B)** F_m_ (open bars) and F_m_′ (striped bars); **(C)** F_v_/F_m_ (open bars) and Y_(II)_ (striped bars); **(D)** (F_t_ – F_o_)/F_o_; **(E)** (F_m_′ – F_*m*_)/F_m_; **(F)** 1-(F_V_/F_*m*_ – Y_(II)_)/(F_V_/F_m_). In each case, 625 nm ML and AL was used. Values represent the average of three biological replicates; standard errors and trendlines are indicated as error bars and dashed lines, respectively.

Besides ETR parameters (Figures 1D–F), PAM fluorometry allowed for monitoring other photosynthetic parameters. These parameters were either derived from fast OJIP transitions (Figure 6) or from traditional (slow) fluorescence induction curves (Figure 7 and Supplementary Figure 6). The shape of the OJIP curves, and especially the fluorescence intensities at the I-step (30 ms), strongly depend on the quality of the growth light (Figure 6A). The cells grown under 465 and 495 nm light showed the highest Chl *a* fluorescence at this step, while cells grown under 687 nm displayed the lowest (Figure 6A). The kinetics (slope) of the corresponding J–I transition showed the same trend (Figure 6B), indicating (i) a more reduced PQ pool in the cultures grown under blue/green light and (ii) an oxidized PQ pool in the 687 nm grown cells. Quantitative analysis of the OJIP kinetics revealed low efficiency of transfer of both PSII (δR_0_) and PQH_2_ trapped electrons (ψR_0_) to PSI acceptors in the blue/blue-green light grown cells and the opposite in the cells grown under 687 nm light (Figures 6C,D). No significant changes in the efficiency of electron transfer from Q_A_^–^ to PQ (ψE_0_) were found (Figure 6E; for definition of OJIP parameters, see Stirbet et al., 2018). These results suggest that the reduced PQ pool in *C. gracile* cells grown under blue/green light was due to a diminished PQH_2_ to PSI electron transfer, while there was no change in the corresponding efficiency of the Q_A_^–^ to Q_B_^–^ electron transfer. In other words, the efficiency of reoxidation of a reduced PQ pool by downstream carriers was lower in these cells.

The dependencies of the initial (F_o_), steady-state (F_t_) and maximal (F_m_ and F_m_′) fluorescence yields on the wavelength of growth light showed similar patterns, with maximal and minimal values in the blue/green and red regions, respectively (Figures 7A,B). The observed high fluorescence yields in blue/green light grown cultures concur well with the high PBS levels in these cells (c.f. Figures 2C, 3A), while the relatively low F_o_ and F_m_ values were in apparent contradiction with the interpretation of fluorescence data (i.e., partial PBS decoupling) in section “Excitation of Photosynthetic Pigment-Protein Complexes” above (see e.g., Acuña et al., 2016; Remelli and Santabarbara, 2018; Santabarbara et al., 2019). However, this could be solved if F_o_ were normalized to the amount of absorbed light quanta and also to pigment content (Supplementary Figure 5). Normalization of F_o_ to the amount of absorbed light quanta (by 625 nm excitation) revealed high values for the 495 nm and, again, for the 687 nm grown cells, in line with the relatively high PBS content in these cultures (Figure 3A and Supplementary Figure 5A). Further, the F_o_/PBP ratios were remarkably high in all cultures grown under orange-red lights, while the 687 nm grown cells showed an about two-fold higher F_o_/Chl ratio as compared to the other cultures (Supplementary Figures 5B,C).

Due to the similar patterns of F_o_ and F_m_, not surprisingly, the calculated F_*v*_/F_m_ values also follow a similar trend, with a maximum at 465 nm (Figure 7C). On the contrary, the corresponding PSII effective quantum yields, Y_(II)_, were the lowest in the blue spectral region and the highest in the red region (Figure 7C). This, again, indicates an easily reducible PQ pool in the red light grown cells.

To separate changes in the fluorescence yields caused by functional changes in the photosynthetic machinery from the influence of potential alterations in the PBS abundance, we calculated normalized differences of the corresponding fluorescence parameters, i.e., (F_*t*_ – F_o_)/F_o_, (F_m_′ – F_m_)/F_m_, and 1-(F_*v*_/F_m_ – Y_(II)_)/(F_*v*_/F_m_) (Figures 7D–F). The analysis revealed high F_*t*_ levels (as compared to F_o_) in the cultures grown under violet to green and also under near-far red light (Figure 7D), which suggests a considerable closure of the PSII reaction centers in these cultures upon AL illumination. The trend was the opposite for the F_m_ vs. F_m_′ and F_*v*_/F_m_ vs. Y_(II)_ parameters, where the red light grown cells showed the highest relative increase upon (625 nm) AL illumination (Figures 7E,F and Supplementary Figure 6), showing, in turn, an effective State 2 to State 1 transition. Interestingly, the cells with a redox imbalance (Figure 6A) showed the highest non-photochemical quenching (NPQ) using 480 nm AL, whose wavelength is the most effective in inducing cyanobacterial NPQ (Supplementary Figure 7; Rakhimberdieva et al., 2004). This suggests an increased abundance of orange carotenoid protein (OCP) in these cultures (see Plohnke et al., 2015) and possibly also the complementary role of short-term light acclimation processes, namely state transitions and blue-light induced OCP quenching, in cyanobacteria (see also Discussion, Kaňa et al., 2012; Bernát et al., 2018).

## Discussion

### Plasticity of the Photosynthetic Machinery

Photosynthetic organisms dynamically adjust the number of light quanta reaching the reaction centers and also the light energy distribution between PSII and PSI. In the absence of such adjustment, the electron transport chain would become under- or oversaturated; resulting in sub-optimal photosynthetic performance or damage of the photosynthetic machinery and other cellular components. During long-term light acclimation cells optimize their light-harvesting antenna size and composition, and PSII/PSI ratios according to incident light conditions to poise the redox state of the electron transport chain, and especially of the PQ pool that transports electrons from PSII to downstream carriers (Fujita et al., 1994; Fujita, 1997; Dietzel et al., 2008). Specific form of long-term light acclimation is cyanobacterial CA, in which CA-capable cyanobacteria adjust the pigment composition in their PBS according to light quality (Tandeau de Marsac, 1977; Sanfilippo et al., 2019; see also Introduction). However, according to literature and our data, CA-incapable cyanobacteria (including *C. gracile* according to current classification) are also able to sense and respond to changing light colors (Fujita et al., 1985, 1987, 1994; Fujita and Murakami, 1987; Fujita, 1997; Fiedler et al., 2004; Bernstein et al., 2014; this study).

In this work we comprehensively studied the light color acclimation of *C. gracile*, a common cyanobacterium living in turbid freshwater shallow lakes (like Lake Balaton or other Central-European lakes), using nine different monochromatic growth lights covering the spectral range from 435 to 687 nm. According to our data, *C. gracile* cells performed great plasticity in terms of their pigment composition, antenna size, and photosystem stoichiometry (Figures 2–4). Oxidation pressure on the intersystem chain via withdrawal of electrons from linear electron transport chain by PSI under orange or red illumination is partially balanced by increasing photosystem II to photosystem I ratios (Figure 4). On the other hand, unfavorable light conditions, where neither chlorophyll nor phycobilisomes absorb light sufficiently, are compensated by an enhanced antenna size and/or increased carotenoid levels (Figures 2, 3).

### Redox Imbalance in *C. gracile* Grown Under Blue and Near Far-Red Light

In spite of the compensatory strategies described above, *C. gracile*, like other cyanobacteria, uses blue light less efficiently, which involves moderate growth rates, reduced cell volumes and low ETR (Figure 1). In the previous work of Luimstra and her co-workers, the less efficient use of blue light in (common) cyanobacteria was explained by a redox imbalance between PSII and PSI, an inherent consequence of PBS-possession of cyanobacteria that are evolutionarily adapted to red-enriched light (Luimstra et al., 2018). Conversely, they explained the fact, why PBS-less marine cyanobacteria, i.e., *Prochlorococcus* species are dominated in deep marine environments that can only be penetrated by blue and green light. We confirmed that hypothesis by PAM-fluorimetry, showing a largely reduced intersystem chain and ineffective photosynthesis in *C. gracile* cells grown under blue light (Figures 6, 7).

Further, we found strong bioenergetic imbalance not only in *C. gracile* cells grown under blue light, but also in cells grown under near far-red (687 nm) light, which is also effectively absorbed by PSI. Surprisingly, orange, red or near far-red growth light triggered a partial excitonic PBS decoupling from photosystems and even from thylakoid membranes, which was the most pronounced in the cells grown under 687 nm light (Figure 4). Although energetic decoupling of the PBS antenna from reaction centers occurs even under normal conditions (Chukhutsina et al., 2015), so far, similar phenomenon could have been induced only by strong light (Tamary et al., 2012). It is quite astonishing that under mild physiological conditions it is also possible to induce such permanent decoupling. This strongly suggests that suboptimal photosynthetic performance of near far-red light grown *C. gracile* cells (Figure 1) is a consequence of a solid redox-imbalance (PQ pool oxidation, which was opposite to PQ pool reduction in blue/green light grown cells). High levels of the photoprotective carotenoid zeaxanthin in cells grown under 687 nm light (Figures 2, 3) support this assumption. Also, despite the 687 grown cells having oxidized PQ pool upon dark acclimation (Figure 6), their intersystem chain turned over-reduced under AL (Figure 7, this result does not contradict results of OJIP measurements that were performed after dark acclimation). The permanent excitonic decoupling of PBS from PSII reaction centers (Figures 4, 5) under weak light suggest (i) a physiological role of such light acclimation process (ii) that might be triggered by a different (path)way, rather than via a local heat transient induced process as shown previously (Stoitchkova et al., 2007; Tamary et al., 2012; see also below).

### Excitonic Decoupling as a Light Acclimation Process

The two major short-term light acclimation processes in cyanobacteria are state transitions and blue-light induced non-photochemical quenching. They occur in minutes and reflect short-term changes in light intensity. Similarly to long-term light acclimation, these processes also attempt to balance the PQ redox state. Regarding state transitions, it is generally assumed that in state 1 (PQ pool relatively oxidized) and state 2 (PQ pool relatively reduced) the photosynthetic machinery displays optimal quantum yields of photosynthesis in light that has a composition favoring its absorption by PSI or PSII, respectively. Different from higher plants and green algae, cyanobacteria are in state 1 upon illumination and in state 2 in the dark or in very low light due to the respiratory electron flow reducing the PQ pool (that is shared by both photosynthesis and respiration, Mullineaux and Allen, 1986). According to the data available, in cyanobacteria both relative energy transfer from PBS to photosystems and distribution of the absorbed light energy between photosystems are regulated by state transitions (Mullineaux and Emlyn-Jones, 2005), although the detailed molecular mechanism of these processes is still unknown.

Also different from higher plants, NPQ in cyanobacteria is pH-independent and can be exclusively induced by “blue” light. A specific carotenoid molecule was shown to play a central role in this process (Rakhimberdieva et al., 2004). This pigment was identified as a hydroxyechinenone or echinenone chromophore in the water soluble OCP (for current reviews see, Kirilovsky and Kerfeld, 2016; Kerfeld et al., 2017), which is bound to PBS and reversibly converted from the (dark-stable) orange form to the (active) red form upon illumination with strong blue light. The red form is essential for the induction of the photoprotective mechanism (Wilson et al., 2008). Experimental data suggest that the quenching center is formed at the level of the PBS core, most likely at allophycocyanin trimmers emitting light at 660 nm (Tian et al., 2011; Jallet et al., 2012).

Our results show that both state transitions and OCP-quenching can occur in *C. gracile* and their contribution to short-term light acclimation processes is largely dependent on the wavelength of growth light: the efficiency of the state transitions was the highest in the cells grown under red light (Figure 7), while OCP-induced NPQ was the maximal in the cultures long-term acclimated either to blue-green or 687 nm light and exhibiting solid redox-imbalance (Supplementary Figures 7,6A). This highlights the increased accumulation of OCP under oxidative stress (Plohnke et al., 2015), possibly in close relation to the essential role of OCP as a singlet oxygen quencher (Sedoud et al., 2014). Moreover, the complementary feature of state transitions and OCP quenching also emphasize the potential interplay between various short-term light acclimation processes (Kaňa et al., 2012; Bernát et al., 2018).

Recently, we have discovered a third type of short-term light acclimation process in cyanobacteria, in which excitonic decoupling of PBSs from PSII reaction centers upon strong illumination plays a central role (Tamary et al., 2012). It was assumed that the process was photon-dose dependent and the mechanism was directly triggered thermally via local heat transients (Stoitchkova et al., 2007; Tamary et al., 2012). In this work we provided evidence that under certain conditions even weak light (i.e., 25 μmol photons m^–2^ s^–1^) can induce such permanent excitonic decoupling in *C. gracile*. Hence, although the role of local heat transients cannot be neglected, excitonic decoupling from the PBS antenna from PSII can also be induced by a redox signal cascade in this cyanobacterium. PAM-fluorimetry data, showing a largely reduced intersystem chain in AL-illuminated *C. gracile* cells grown under near far-red light (Figure 7), supports this hypothesis. High cellular zeaxanthin level (Figure 3), which has a general photoprotective role in oxygenic photoautotrophs is also in line with this assumption. In this respect, PBS decoupling induced under weak near far-red light may imply a new type of light acclimation process.

## Data Availability Statement

The original contributions presented in the study are included in the article/Supplementary Material, further inquiries can be directed to the corresponding author.

## Author Contributions

GB proposed the research questions, designed the study, performed experiments, contributed to the results analyses, and wrote the manuscript. TZ performed and analyzed cell counting and photosynthesis activity measurements and contributed to growth experiments and analyses. EK and LK performed the HPLC measurements and analyzed the results. GS performed the confocal microscopy and analyzed the results. LV and BS designed the lighting apparatus and contributed to the growth experiments. OP and VT supervised the project. All authors contributed to the article and approved the submitted version.

## Conflict of Interest

The authors declare that the research was conducted in the absence of any commercial or financial relationships that could be construed as a potential conflict of interest.
