# Supplementary material for: Photomorphogenesis in the Picocyanobacterium Cyanobium gracile Includes Increased Phycobilisome Abundance Under Blue Light, Phycobilisome Decoupling Under Near Far-Red Light, and Wavelength-Specific Photoprotective Strategies
Source: Front Plant Sci. 2021 Mar 18;12:612302. doi: 10.3389/fpls.2021.612302 (PMC8012758; doi:10.3389/fpls.2021.612302)
Supplement: Supplementary file 1 [file Data_Sheet_1.docx]

**Supplementary information**

**Photomorphogenesis in the picocyanobacterium *Cyanobium gracile* includes increased phycobilisome abundance under blue light, phycobilisome decoupling under near far-red light, and wavelength-specific photoprotective strategies**

Gábor Bernát^a,b,*^, Tomáš Zavřel^c^, Eva Kotabová^b^, László Kovács^d^, Gábor Steinbach^e,f^, Lajos Vörös^a^, Ondřej Prášil^b^, Boglárka Somogyi^a^ and Viktor R. Tóth^a^

^a^*Centre for Ecological Research, Balaton Limnological Institute, Hungarian Academy of Sciences, Klebelsberg Kuno u. 3., 8237 Tihany, Hungary*

^b^*Centre Algatech, Institute of Microbiology, Academy of Sciences of the Czech Republic, Opatovicky mlyn, 379 81 Třeboň, Czech Republic*

^c^*Global Change Research Institute, Academy of Sciences of the Czech Republic, Bělidla 986/4a, 603 00 Brno, Czech Republic*

^d^*Institute of Plant Biology*, ^e^*Institute of Biophysics,* and *^f^Cellular Imaging Laboratory*, *Biological Research Center*, *Eötvös Loránd Research Network, Temesvári krt. 62., H-6726 Szeged, Hungary*


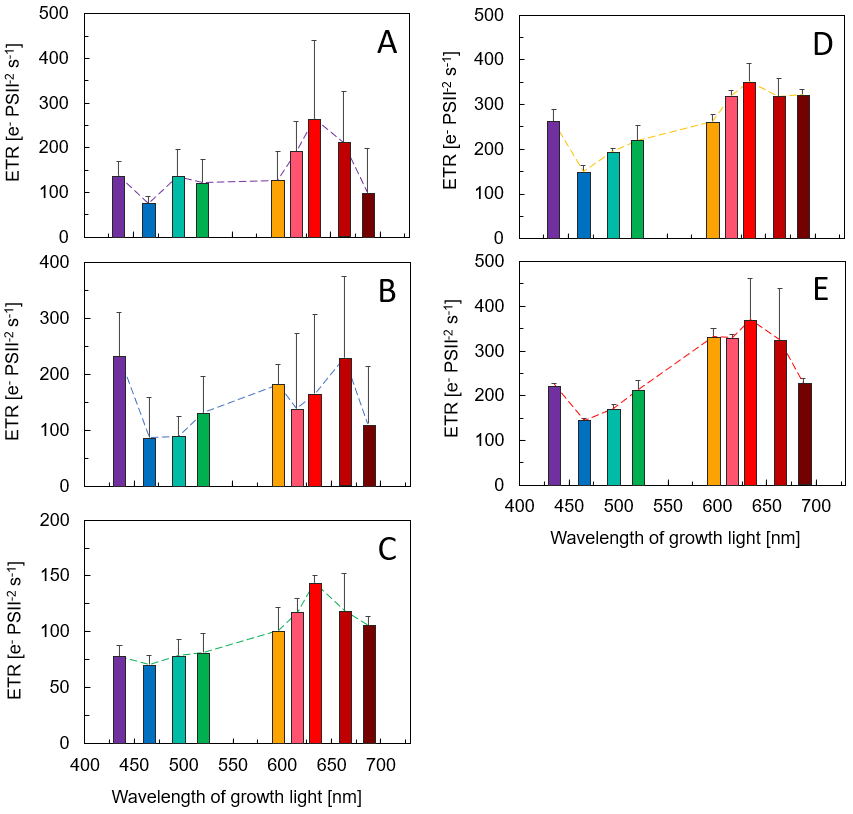


**Figure S1** Effect of actinic light (AL) and measuring light (ML) wavelengths on electron transport rates (ETR) as determined by saturating pulse (SP) analysis during fluorescence induction measurements. During the recordings, both AL and ML wavelengths were set to 440 nm (A), 480 nm (B), 540 nm (C), 590 nm (D) and 625 nm (E). The data represent averages of three biological replicates; error bars represent standard deviations. For details see section 2.3 in Materials and Methods.

**
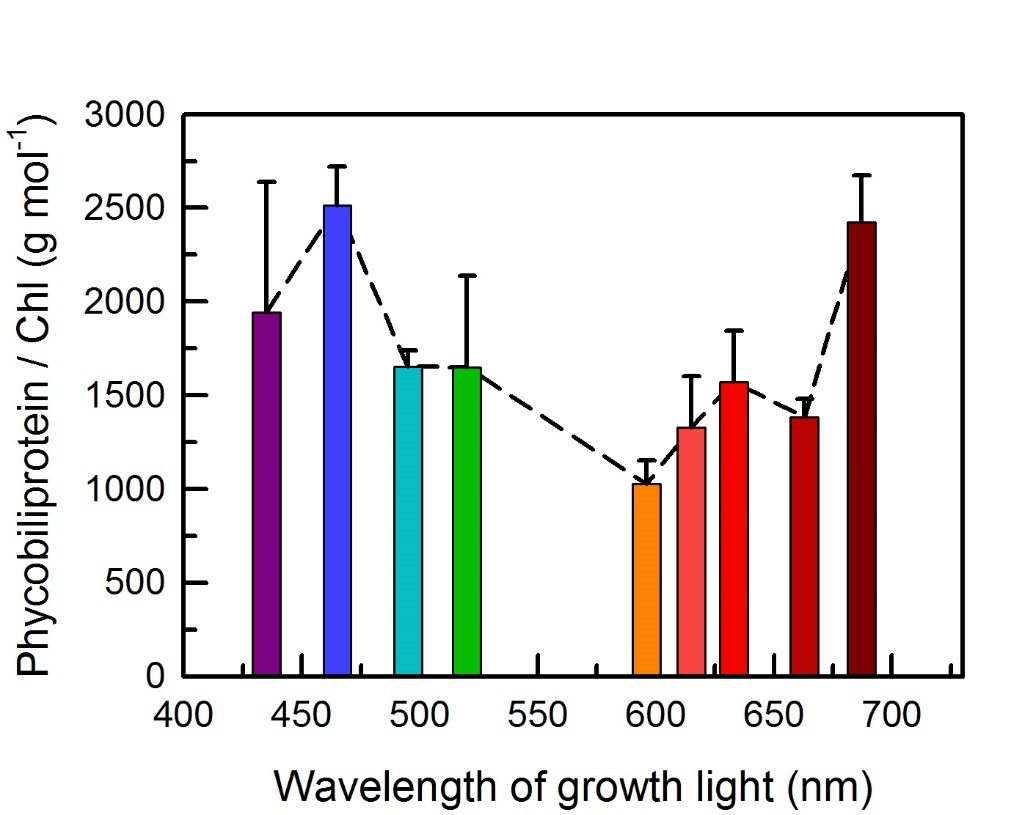
**

**Figure S2** Phycobiliprotein to Chl *a* ratios in *C. gracile* sp. ACT 1026 cultures grown under monochromatic lights. Total phycobiliprotein content of the cultures was determined based on the absorbance spectra shown in Fig. 2B, while the level of Chl *a* was determined by HPLC. Data are expressed as mean ± standard error (n=3). Dashed lines represent trend lines.

**
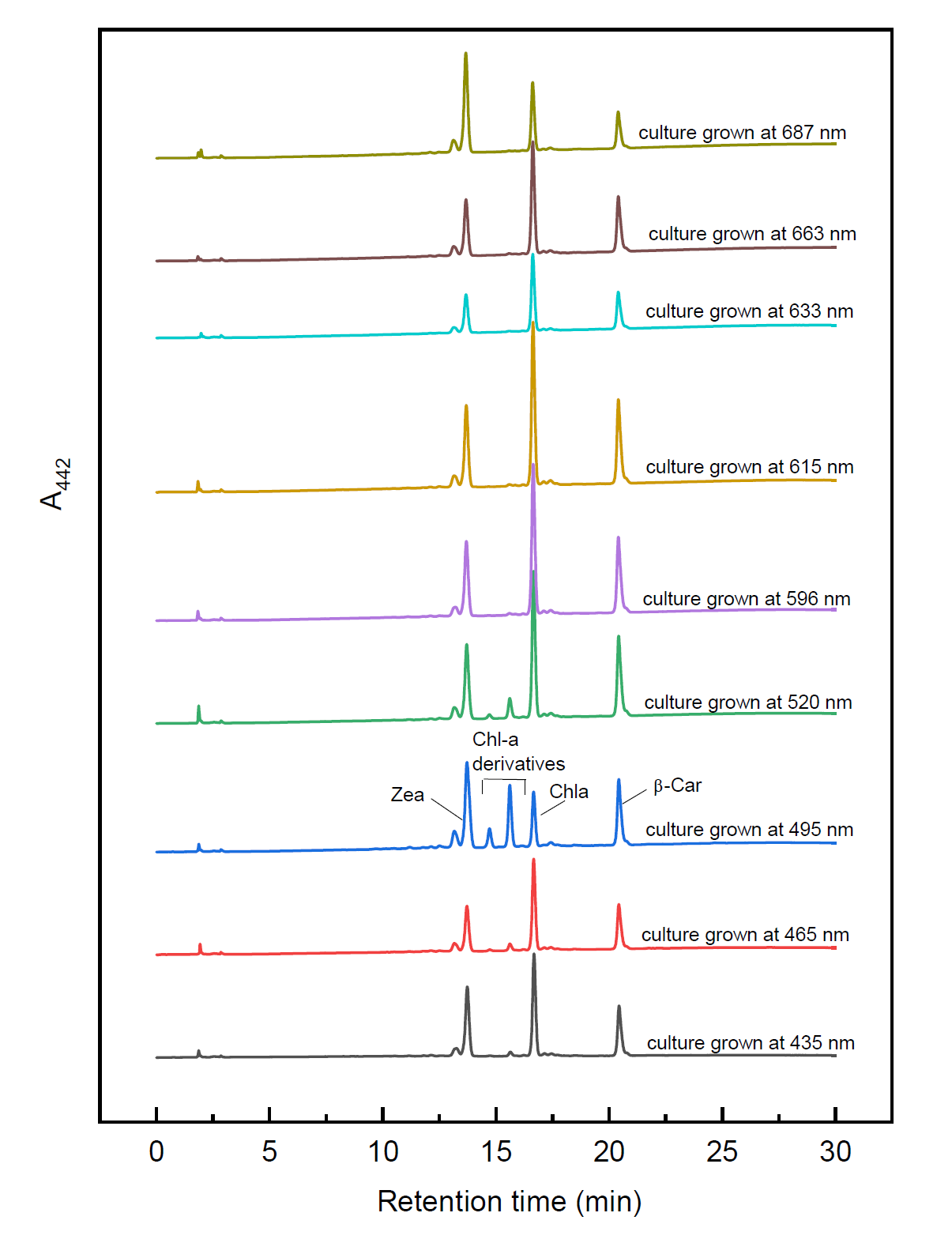
**

**Figure S3** HPLC chromatograms of acetonic extracts from *C. gracile* cells grown under monochromatic lights (as indicated above each line). Major peaks at the chromatogram of the 520 nm-grown cells are labelled as: Zea = zeaxanthin; Chl *a* = chlorophyll *a*; β-Car = β-carotene. Chl *a* derivatives (Chl *a* allomers) are present in a significant amount in the 495 and 520 nm grown cells. The chromatograms were recorded at 442 nm and represent a typical record under each particular wavelength (out of three measurements in total that showed high similarity at both qualitative and quantitative level).


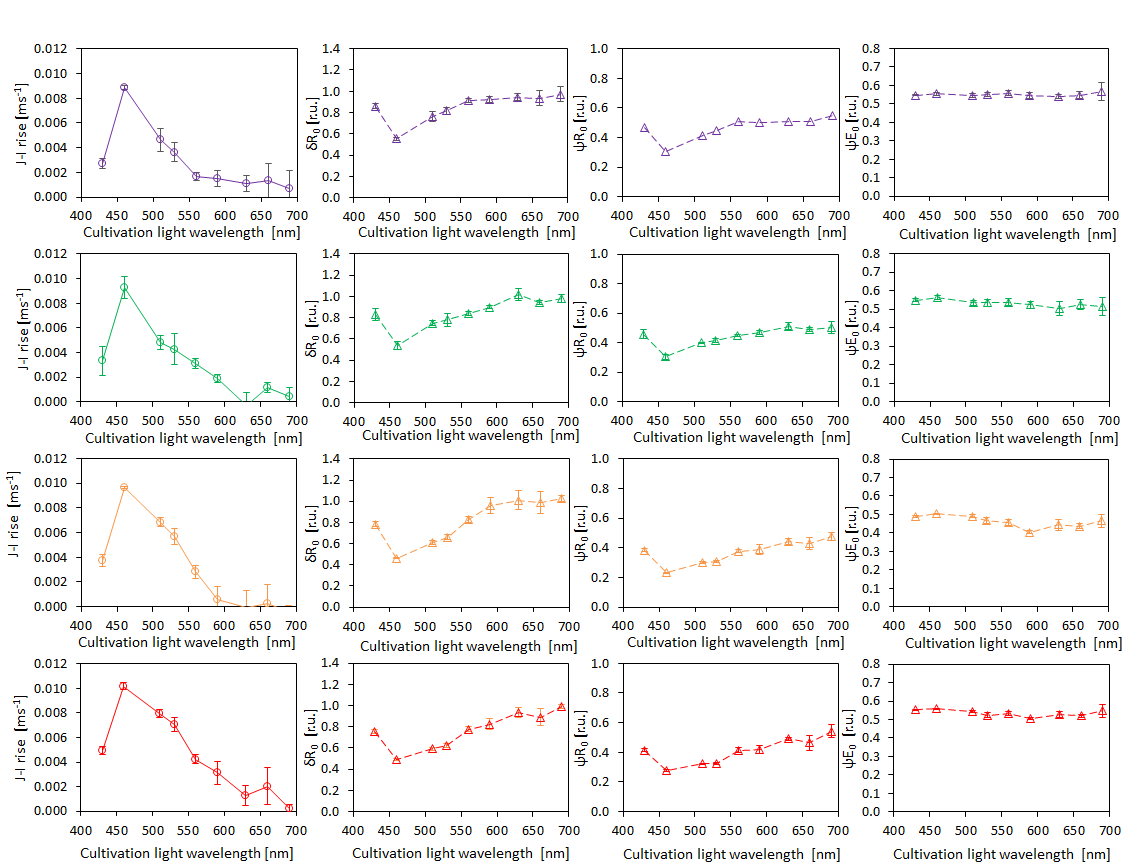


**Figure S4** Effect of measuring light (ML) wavelength on traces of parameters derived from OJIP measurements (for details see section 2.3 in Materials and Methods). Data represent averages of three biological replicates; error bars represent standard deviations. ML wavelengths were set to 440 nm (violet lines), 540 nm (green lines), 590 nm (orange lines) and 625 nm (red lines). The parameters included rate of fluorescence increase during the J-I phase (J-I rise), efficiency with which an electron from PQH_2_ is transferred to final PSI acceptors (δR_0_), efficiency with which a PSII-trapped electron is transferred to final PSI acceptors (ψR_0_) and efficiency with which a PSII-trapped electron is transferred from Q_A_^-^ to PQ (ψE_0_), according to (Stirbet et al. 2018).

**
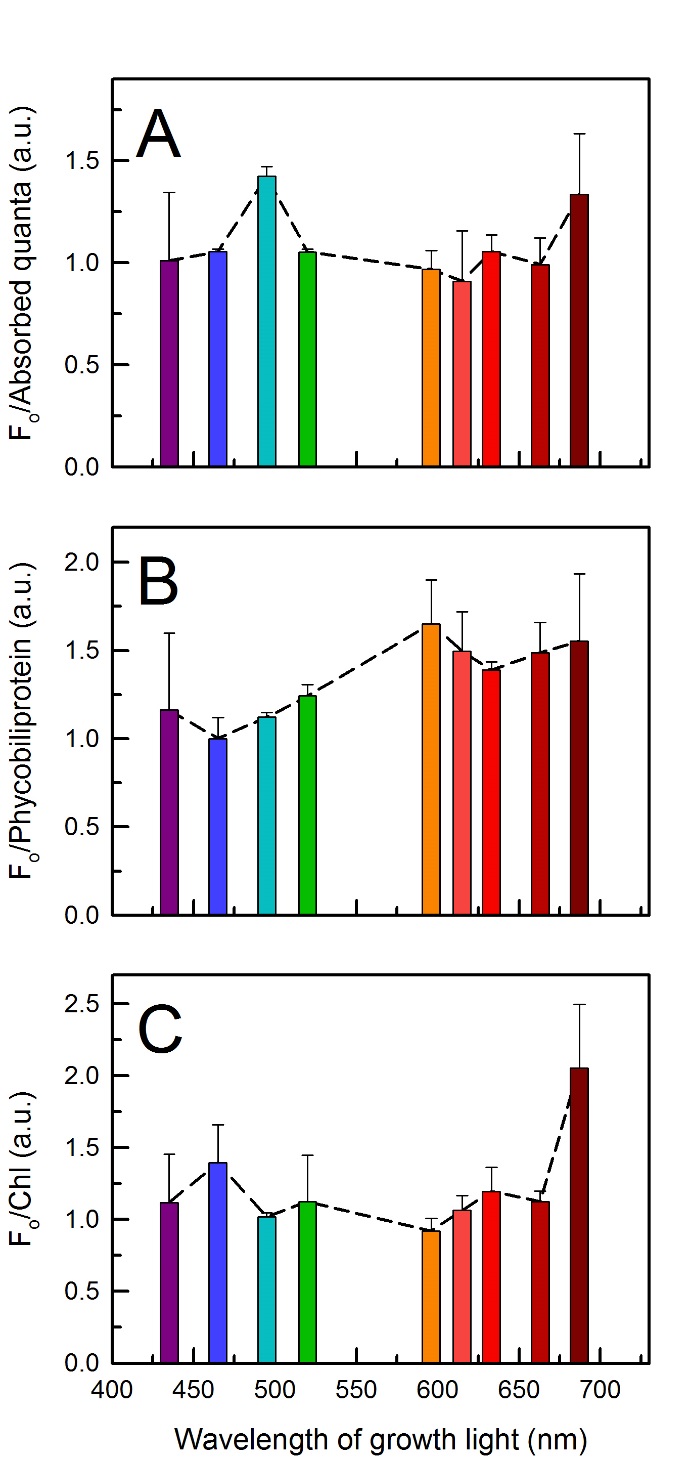
**

**Figure S5** Normalized initial fluorescence levels (F_o_) of *C. gracile* cells grown under monochromatic lights (as detailed in Fig. 1A). F_o_ levels, determined by 625 nm ML, was normalized to the amount of absorbed light quanta (A), phycobiliprotein content (B) and Chl content (C). The amount of absorbed light quanta on panel A was determined as $\int E\left( \right)A\left( \right)d$, where $E\left( \right)$ and $A\left( \right)$ represent the emission of the 625 nm chip-on-board LED emitter of the MC-PAM and the absorbance of the corresponding culture at a certain wavelength (see Fig. 2B), respectively. Values represent the average of three biological replicates; standard errors and trendlines are indicated as error bars and dashed lines, respectively.


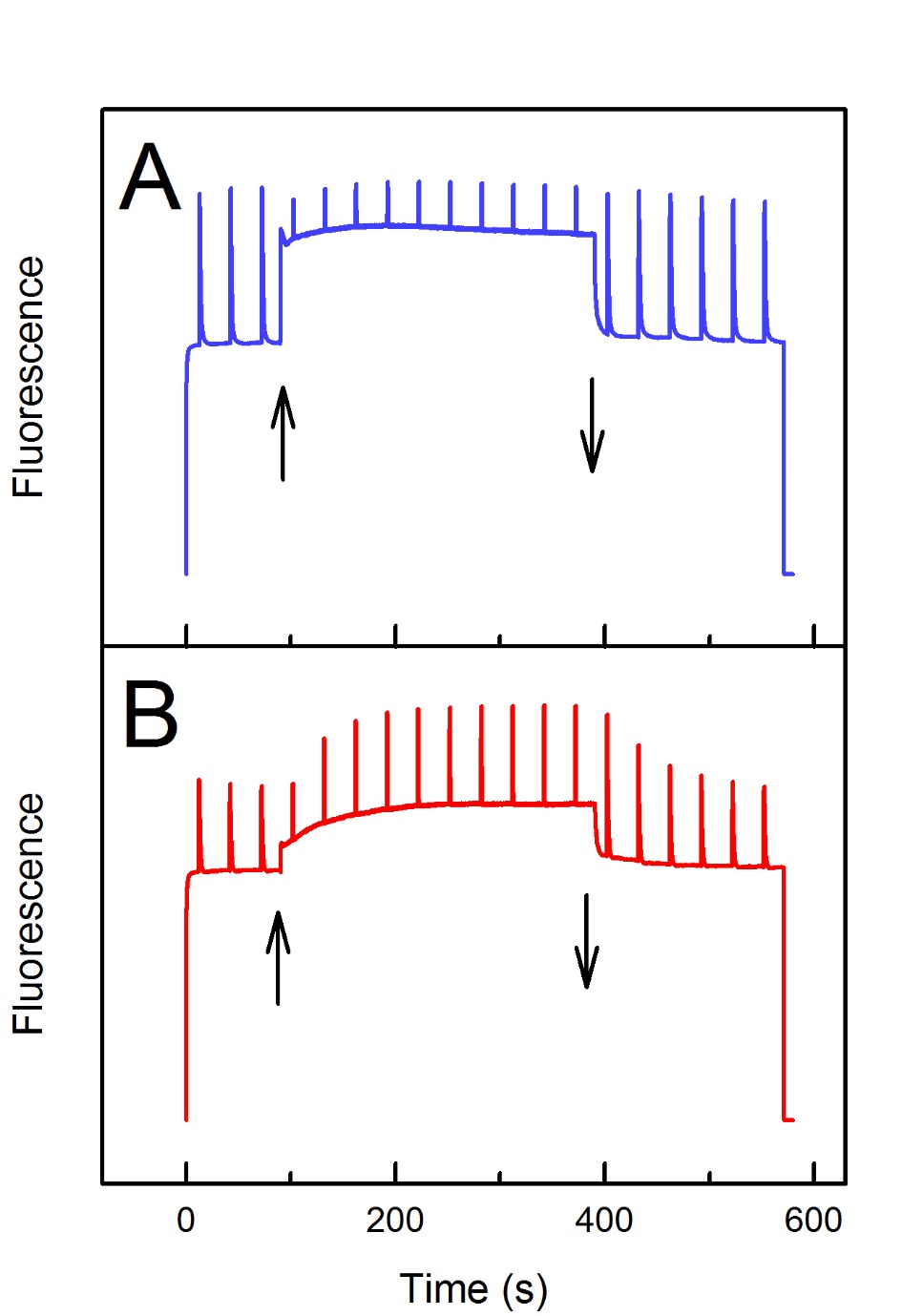


**Figure S6** Fluorescence induction curves of *C. gracile* cells grown under 465 nm (A) or 633 nm (B) growth light using 625 nm AL and ML to probe state transitions (for details see section 2.3 in Materials and Methods). Upward and downward arrows represent the AL on and off, respectively.


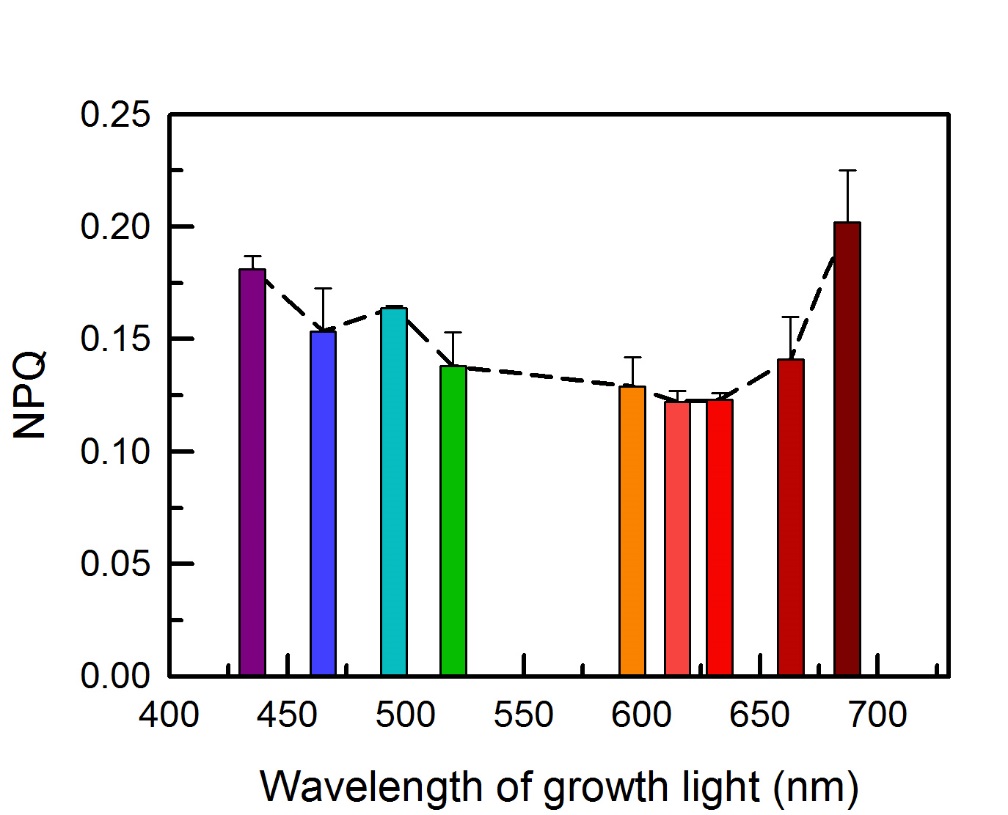


**Figure S7** Non-photochemical fluorescence quenching (NPQ) as detected during rapid light curves using 480 nm AL and ML. NPQ values were calculated using the saturating pulse at the terminal step of recordings with 1300 μmol photons m^-2^ s^-1^ AL. Data represent averages of three biological replicates; error bars represent standard deviations. For details see section 2.3 in Materials and Methods. The equation for NPQ calculation was derived from Serôdio et al. (2006).

**References:**

Serôdio J, Vieira S, Cruz S, Coelho H (2006) Rapid light-response curves of chlorophyll fluorescence in microalgae: relationship to steady-state light curves and non-photochemical quenching in benthic diatom-dominated assemblages. Photosynth Res 90: 29–43

Stirbet, A., Lazár, D., Kromdijk, J. Govindjee, 2018, Chlorophyll *a* fluorescence induction: Can just a one-second measurement be used to quantify abiotic stress responses? Photosynthetica 56: 86-104.
